# Trends and determining factors associated with adherence to antiretroviral therapy (ART) in Cameroon: a systematic review and analysis of the CAMPS trial

**DOI:** 10.1186/1742-6405-9-37

**Published:** 2012-12-19

**Authors:** Lawrence Mbuagbaw, Lehana Thabane, Pierre Ongolo-Zogo, David Yondo, Stephen Noorduyn, Marek Smieja, Lisa Dolovich

**Affiliations:** 1 Centre for Development of Best Practices in Health (CDBPH), Yaoundé Central Hospital, Avenue Henri Dunant, Messa, PO Box 87, Yaoundé, Cameroon; 2 Department of Clinical Epidemiology and Biostatistics, McMaster University, Hamilton, ON, Canada; 3 Biostatistics Unit, Father Sean O'Sullivan Research Centre, St Joseph’s Healthcare, Hamilton, ON, Canada; 4 St. Joseph's Healthcare Hamilton, Hamilton, ON, Canada; 5 Department of Family Medicine, McMaster University, McMaster Innovation Park, Hamilton, ON, Canada

**Keywords:** Adherence, Antiretroviral therapy, Cameroon, Reminder methods, CAMPS

## Abstract

**Background:**

The benefits of antiretroviral therapy (ART) cannot be experienced if they are not taken as prescribed. Yet, not all causes of non-adherence are dependent on the patient. Having to pay for medication reduces adherence rates. Non- adherence has severe public health implications which must be addressed locally and globally. This paper seeks to describe the trends in adherence rates reported in Cameroon and to investigate the determinants of adherence to ART in the Cameroon Mobile Phone SMS (CAMPS) trial.

**Methods:**

We conducted a systematic review of electronic databases (PubMed, Google Scholar, Web of Science, CINAHL, EMBASE and PSYCINFO) for publications on adherence to ART in Cameroon (from January 1999 to May 2012) and described the trend in reported adherence rates and the factors associated with adherence. Data were extracted in duplicate. We used multivariable analyses on the baseline data for 200 participants in the CAMPS trial to determine the factors associated with adherence in four models using different measures of adherence (more than 90% or 95% on the visual analogue scale, no missed doses and a composite measure: 100% on the visual analogue scale, no missed doses and all pills taken on time).

**Results:**

We identified nine studies meeting our inclusion criteria. Adherence to ART in Cameroon has risen steadily between 2000 and 2010, corresponding to reductions in the cost of medication. The factors associated with adherence to ART in Cameroon are grouped into patient, medication and disease related factors. We also identified factors related to the health system and the patient-provider relationship. In the CAMPS trial, education, side effects experienced and number of reminder methods were found to improve adherence, but only using multiple reminder methods was associated with better adherence in all the regression models (Adjusted Odds Ratio [AOR] 4.11, 95% Confidence Interval [CI] 1.89, 8.93; p<0.001; model IV).

**Conclusions:**

Reducing the cost of ART is an important aspect of ensuring adequate adherence rates. Using multiple reminder methods may have a cumulative effect on adherence to ART, but should be investigated further.

## Background

The advent of antiretroviral medication as a treatment for HIV is one of the most celebrated advances in medicine [1]. Antiretroviral therapy (ART) reduces viral loads to undetectable levels, and dramatically decreases morbidity and mortality. Decreasing serum viral load with antiretroviral medication also reduces mother to child transmission rates [2,3]. However, viral replication still occurs in reservoirs like lymph node or gastrointestinal T-cells even when viral loads are undetectable [4,5]. As such, good adherence is necessary to maintain prolonged viral suppression [5]. Non-adherence to HIV medication is a major reason for treatment failure, the development of resistant strains, and increased costs [6]. It is also one of the key predictors or determinants of success in the management of HIV/AIDS and the progression to AIDS and death [7,8]. Non-adherence reduces the immunologic potential of ART and is associated with drops in CD4-positive-T-lymphocyte counts [9-11]. Non-adherence has also been found to be associated with increased hospitalisations and longer hospital stays [10,12]. Initial worries in the scientific community about non-adherence in sub-Saharan Africa were dispelled when research findings demonstrated similar or higher rates of adherence than in the developed world [13]. However, only a third of people living with HIV (PLHIV) take their medication as prescribed [1]. Non-adherence leads to increased viremia which might lead to increased transmissibility and consequently a higher incidence of HIV. High risk activity may also lead to the transmission of resistant strains to newly infected individuals, thereby reducing their therapeutic options [14,15]. Adherence to ART has clinical and public health implications, and should therefore be addressed on global and local levels.

### Defining adherence

Adherence can be narrowly defined as the “extent to which patients take medications as prescribed by their health care providers” [16], or more broadly as the “the extent to which a person’s behaviour—taking medication, following a diet, or executing lifestyle changes, corresponds with agreed recommendations from a health care provider” [1]. The former definition refers to compliance with medication as prescribed by the provider while the latter goes beyond medication to include all the recommendations jointly agreed upon by both provider and client to improve health care. The latter definition also highlights or acknowledges additional factors such as diet or lifestyle which may affect adherence. It also recognises that the client may not agree with the recommendations of the health care provider. Such factors are even more relevant in HIV where dietary restrictions are required for the uptake of certain medications [17,18] and that certain lifestyles (e.g. substance abuse) can lead to non- adherence to ART [19]. In this paper, all degrees of adherence which are not optimal are referred to as non-adherence.

The factors associated with adherence to ART exist in many categories, defined in literature [1,16,19]:

*Patient factors* such as substance abuse, being male (i.e. gender), depression, lower levels of education, lack of self efficacy, extreme anxiety, extreme pain, no change in health status despite ART and non-white race are significantly associated with non-adherence [1,19].

*Medication factors* like dose frequency, pill burden, type of drug, inability to take medication when away from home, food requirements, side effects are also responsible for less than optimal adherence [1,19]. In other words the complexity of the regimen and its side-effects are associated with non-adherence.

*Providerrelated factors* such as a poor patient-health care provider relationship can affect the patient’s overall satisfaction and trust in the provider. The quality of these relationships is significantly associated with better adherence [1,20,21].

*Disease characteristics*, notably stage and duration of HIV infection, symptoms experienced and the presence of opportunistic infections play a role in adherence to ART. HIV related symptoms like nausea may impede a patient from swallowing pills. Some studies report that patients who have experienced an opportunistic infection tend to be more adherent than those who have not [1,22,23].

*Clinical setting and health system factors* may influence use of services and adherence [19].

### Adherence to ART in Cameroon

Cameroon has one of the highest rates of HIV in west and central Africa, with 5.3% of the adult population living with HIV. Of these, only 30% are receiving ART [24]. Adherence to ART in Cameroon has changed over the years for a multitude of reasons. Health system changes like the decentralization of HIV treatment centres and the subsidy of costs of medication and testing over the years have contributed to the variations in the reported adherence rates. A substantial reduction in costs occurred in 2004 [25] and ART has been provided for free since 2007 [26]. As a result, the designs and the contexts of the studies reporting rates of adherence to ART in Cameroon also vary. The set of similar and varying contextual factors in Cameroon make it a worthwhile case study to examine adherence to ART and learn lessons that could be applied across the country. Adherence rates in Cameroon have been measured using patient reports, pharmacy refill data, attendance at scheduled visits and blood testing [25-35]. These different methods have spawned adherence rates varying from 10.1% to 97.5%, between 2000 and 2010. Faced with these enormous discrepancies, we sought to use data from a recent trial [36] to describe adherence to ART and the associated determining factors. Adherence rates are not only a predictor of treatment success, but also an indication of how many resources should be invested in adherence enhancement research and practice. This cannot be done without stable estimates for adherence or a comprehensive list of locally relevant determinants.

Additionally, in a changing world where new methods and technologies arise to improve adherence rates, and levels of stigma are declining, there may be a shift in patients’ attitudes and practices related to adherence.

The objectives of this paper are:

•to review the literature on adherence in Cameroon with specific emphasis on ART adherence rates and its correlates or determinants, and

•to report the factors associated with adherence to ART at baseline in the Cameroon Mobile Phone SMS trial (CAMPS;36).

## Methodology

### Literature review of ART adherence rates in Cameroon

We searched electronic databases (PubMed, Google Scholar, Web of Science, CINAHL, EMBASE and PSYCINFO) for publications on adherence to ART in Cameroon (from January 1999 to May 2012) using appropriate combinations of keywords such as *Cameroon*, *Cameroun* (*French*), *adherence*, *compliance*, *antiretroviral therapy*, *antiretroviral treatment* and *HIV*. ART was introduced in Cameroon in 1999 [37]. No language restrictions were set. Citations were screened and full text obtained if they reported any measure of adherence to ART in Cameroonian subjects. Completeness and adequacy of reporting in the selected studies were assessed in duplicate by noting their compliance to the 22-item Strengthening the Reporting of Observational Studies in Epidemiology (STROBE) checklist [38]. Data were also extracted in duplicate: citation details; year study was conducted, city in which study was conducted, adherence rate, method of measure of adherence, study design, sample size and the cost of ART at the time. Discrepancies were resolved by discussion. We examined the evolution of adherence rates taking into account the above characteristics. We also identified the reported factors associated with adherence and classified them as patient variables, medication variables, patient-provider relationship and health system characteristics. These categories have previously been identified in literature [16,19]. No statistical pooling was performed. The factors associated with adherence were extracted only if they resulted from multivariable analyses. The reported odds ratios and p-values were used to confirm the associations reported.

### Adherence to ART in CAMPS

We conducted a cross-sectional analytical study based on the baseline data for the Cameroon Mobile Phone SMS (CAMPS) trial [36]. Data were collected from a subset of patients (eligible for the trial) aged 21 or older, who owned mobile phones and who agreed to take part in the trial. The independent variables included socio-demographics like age (in years), gender (male or female), level of education (none and primary or secondary and university), disclosure of status to family members (family aware or not aware of HIV status); clinical features like presence or absence of an opportunistic infection, Centers for Disease Control and Prevention (CDC; [39]) classification (categorised as AIDS defining condition or not), regimen (first line or second line), duration on treatment (in months); the number of drug side-effects experienced and the number of recall methods used. All the independent variables were those reported in literature and collected for the CAMPS trial [36]. The dependent variables were adherence measured using a visual analogue scale (0–100), self report on the number of missed doses and whether the doses were taken on time in the week preceding the interview. We also designed a composite measure of adherence based on all three measures, which we dichotomized as adherent (VAS=100; number of missed doses=0; all doses taken on time=yes) and non-adherent (VAS<100; missed doses >0; all doses taken on time=no).

We performed multivariable logistic regression with adherence (dependent variable) dichotomized at the VAS cut-offs of 90 and 95% (Model I and II). These cut-off points represent the levels of adherence at which sustained viral suppression can occur [5], and those often used in literature [40,41]. We also analyzed the data using the self reports of adherence as the dependent variable, in which case one or more missed doses was considered as non-adherence and no missed dose was considered as good adherence (Model III). We repeated these analyses using the composite measure of adherence (Model IV). The interaction between independent variables was investigated based on associations reported in the general literature and specifically for Cameroon. Variables were introduced into the model in blocks defined by the following categories: socio-demographic, disease-related and medication-related. Model fit was assessed using the Hosmer and Lemeshow goodness of fit statistic [42].

Data were analysed using Predictive Analytic Software (PASW) Version 17.0 (SPSS, Inc., 2009, Chicago, IL, USA). Statistical significance was set at alpha= 0.05. Adjusted odds ratios (AOR), 95% confidence intervals (CI) and p-values are presented. The forest plot was drawn using Stata Statistical Software (STATA) Release 12 (StataCorp LP, 2011, College Station, TX, USA).

## Results

### Literature review

**Figure 1:**
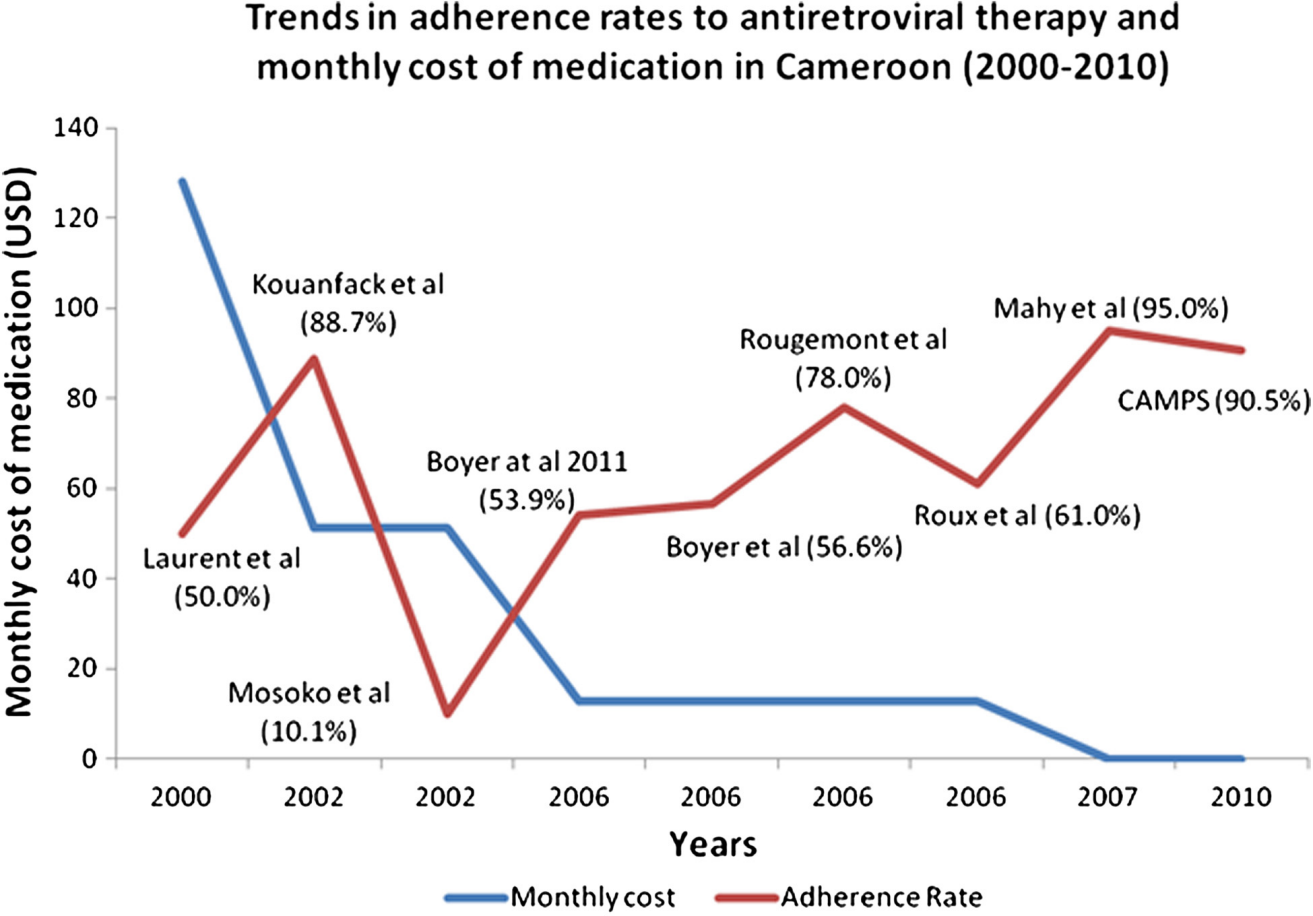
Trends in adherence rates to antiretroviral therapy and monthly cost of medication.

We identified 9 studies conducted between 2000 and 2010 which reported adherence rates in Cameroon. The mean number of reported items on the STROBE checklist was 19.7 (standard deviation [SD] 1.38). Data from the CAMPS trial was included in the analysis [36]. Five of them were conducted in Yaoundé, the capital city of Cameroon. The characteristics of these studies are reported in Table 1. We observed an increasing trend in adherence rates over time, which corresponds to the reductions in cost of ART (Figure 1). Two studies were outliers to this quasi-linear relationship. The first was Kouanfack *et al*. [27], which reported high adherence rates (88.7% and 97.5%, using biological markers and self report respectively). This study was a drug trial and participants received medication free of charge at a time when medication costed 51.2 USD/month. The second, Mosoko *et al*. [25], reported very low adherence rates (10.1% using hospital records of the number of scheduled clinic visits attended). These data may be affected by inaccuracies in hospital records (incompleteness was handled using imputation techniques); participants who lived far away were sometimes given medication for more than one month; patients who lived in the nearby city of Douala had other ART opportunities and this measure (scheduled visits) considers all deaths and lost to follow-up as non-adherent. Ideally, the cases of death should be excluded from the denominator. The other study using attendance of scheduled visits [29] also reported low adherence rates (<50%).

**Table 1:** Characteristics of studies reporting adherence to ART in Cameroon

**Reference**	**Year**	**City**	**Study design (sample size)**	**Adherence rate**	**Method**	Cost of ART/month for that year in USD*
**Laurent et al.**	2000-2003	Douala	Cross-sectional retrospective (788)	<50% ^μ^	Scheduled visits attended	128
**Kouanfack et al.**	2002-2003	Yaoundé	Prospective cohort (60)	88.7%, (97.5%)	Biological markers, (Self Report)	51.2 (free for participants)
**Mosoko et al.**	2002-2005	Limbe	Cross-sectional retrospective ^α^ (2920)	10.1%	Scheduled visits attended	51.2
**Boyer et al.**[28]	2006	Yaoundé	Cross-sectional (532)	56.6%	Self Report ^β^	12.8
**Rougemont et al.**	2006	Yaoundé	Prospective cohort (312)	64% (78%)	Pharmacy data (Self report)	12.8
**Boyer et al.**[32]	2006-2007	Multiple locations	Cross-sectional (3151)	53.9%	Self report ^β^	12.8
**Roux et al.**	2006-2008	Yaoundé	Prospective cohort (401)	61-73%	Self report ^β^	12.8
**Mahy et al.**	2007-2008	Tokombere	Cross-sectional (56)	95% (80%)	self report ^β^ (Pharmacy data)	0
**Mbopi-Keou et al.**	2010	Dschang	Cross-sectional (356)	80.2% (48.7)	Self report ^β^ (Pharmacy data)	0
**CAMPS**	2010	Yaoundé	Cross-sectional (200)	90.5%	Self report ^β^	0

*Costs vary per regimen, the highest costs are reported.

^α^ Cross- sectional analysis of previously collected data without planning for needs of an investigation.

^β^ All methods in which all information used to measure adherence was obtained by interviewing the participant with either one question or a series of questions.

^μ^ 50% was imputed to represent the trend graphically.

### Determinants of, and factors associated with ART adherence

In addition to the nine papers that reported adherence rates, we identified two papers which reported on the determinants of adherence to ART. These studies were conducted in cross-national cohorts of patients from Cameroon, the Democratic Republic of Congo and Burundi (Table 2). These papers were provided additional information on factors associated with adherence [34,35]. They were not used in the trend analysis.

**Table 2:** Factors associated with adherence to ART in literature reports from Cameroon

**Factors**	**Reference (findings)**
**Patient variables**	
Female gender	Rougemont et al. (↑)
Age>49 years	Newman et al.* (↑); Freeman et al.**(↑)
High monthly income	Rougemont et al. (↓); Boyer et al. ([28] ; ↑)
Education	Freeman et al. (↑)*
Binge drinking	Boyer et al. ([32] ; ↓); Roux et al. (↓); Newman et al.(↓)*
Drug use	Freeman et al.(↓)*
Tobacco use	Freeman et al.(↓)*
Lack of family support for adherence	Boyer et al. ([32]; ↓);
Experiencing discrimination and stigma	Boyer et al. ([32]; ↓);
Positive perception of treatment	Roux et al. (↑)
Being transferred-in to HIV clinic	Mbopi-Keou (↑)
**Medication variables**	
Switching regimen	Boyer et al. ([32]; ↓);
High motivation	Roux et al.(↑)
Using a reminder method	Roux et al.(↑)
**Patient Provider Relationship**	
Satisfaction with information provided by physician	Roux et al.(↑)
**Disease characteristics**	
Advanced stage of disease	Rougemont et al. (↓); Roux et al.(↑)
Increased duration on medication	Roux et al.(↓); Freeman et al.(↑)*Mbopi-Keou (↑)
Higher CD4^¥^ count at initiation of ART ^β^	Mbopi-Keou (↑)
**Health System/clinic Characteristics**	
Cost of care/Having to pay for care	Mosoko et al.(↓), Boyer et al. ([32] ; ↓) ; Boyer et al. ([28] ; ↓) Laurent et al. (↓)
Increased distance from clinic	Mosoko et al. (↓)
Large hospital size	Boyer et al. ([32]; ↓);
No task shifting from physician to other staff	Boyer et al. ([32]; ↓);

↓Reduces adherence; ↑increases adherence; * Cohort included participants from Cameroon, Burundi and the Democratic Republic of Congo; ** Cohort included females from Cameroon, Burundi and the Democratic Republic of Congo; ^¥^ CD4-positive-T-lymphocyte; ^β^ Antiretroviral therapy.

These factors were found to reduce adherence to ART: binge drinking, drug use, tobacco use, lack of family support, experience of stigma, switching regimen, advanced stage of disease, cost of care, distance from clinic, large hospital size and no task shifting from physician to other staff [25,28,30-35].

These factors increased adherence to ART: Female gender, age greater than 49 years, higher levels of education, positive perceptions of treatment, high motivation, using reminder methods, satisfaction with information provided by physician, higher CD4 count at initiation of ART, and being transferred-in from another clinic [30,31,33-35].

High monthly income [31,32] and increased duration on medication [30,33,35] were both reported to either increase or reduce adherence.

### Factors associated with adherence to ART at baseline in the CAMPS Trial

In this sample of 200 adults, one fifth was older than 50 years, three-quarters were female, 98% had at least primary education, and 90% reported that their families were aware of their HIV status. The rest of their characteristics are reported in Table 3. All the factors associated with adherence that were collected in this data set were used in the regression analysis. The baseline data for participants in the CAMPS trial are reported in Table 3.

**Table 3:** Baseline characteristics of participants in the CAMPS trial

**Variable**	**Statistic**
**Age (years) : mean (SD)**^**α**^	40.1 (10.10)
**21-49**	157 (79.3)
**50+**	41 (20.7)
**Gender: n (%)**	
**Female**	147 (73.5)
**Level of education: n (%)**	
**None or primary**	78 (39.0)
**Secondary or university**	122 (61.0)
**Family aware of HIV status: n (%)**	180 (90.0)
**Presence of an opportunistic infection: n(%)**	62 (31.0)
**BMI : mean (SD)**^**β**^	25.2 (4.00)
**Underweight**	4 (2.2)
**Normal**	90 (48.6)
**Overweight**	91 (49.2)
CDC* classification - AIDS defining illness§:n (%)	146 (73.0)
**Regimen: n (%)**^**δ**^	
**First line**	179 (90.9)
**Second line**	18 (9.1)
**Duration on ART (months): median (Q1, Q3)**^**α**^	28.5 (9.0, 48.0)
**CD4 (cells per mm**^**3**^**): median (Q1, Q3)**	336.0 (200.5,487.7)
**Adherence**	
**Visual Analogue Scale: mean (SD)**^**ε**^	90.5 (12.76)
**Number of missed doses: mean (SD)**^**μ**^	1.0 (0.00)
**No missed doses: n (%)**	127 (65.1)
**Treatment taken on time: n (%)**	108 (54.0)
**Reasons for missing doses: n (%)**	
**Forgot**	54 (27.0)
**Out of home**	23 (11.5)
**Out of tablets**	7 (3.5)
**Too busy**	8 (4.0)
**Side effects**	2 (1.0)
**Other reason**	5 (2.5)
**Medication side effects experienced: n (%)**	
**None**	127 (63.5)
**One**	43 (21.5)
**Two**	30 (15.0)
**Reminder methods: n (%)**^**¥**^	
**None**	49 (25.7)
**One**	125 (65.4)
**Multiple**	17 (8.5)

SD: standard deviation; BMI: body mass index; *Centres for Disease Control, § =CDC classifications: A3, B3, C1, C1, C3 [39]; α= 2missing; δ=3 missing; β=15 missing; ε= 10 missing ¥= 9missing; μ= 5 missing.

The reminder methods reported were: personal verbal reminders by individuals, phone alarms, meal times, timing with TV shows and watches.

**Figure 2:**
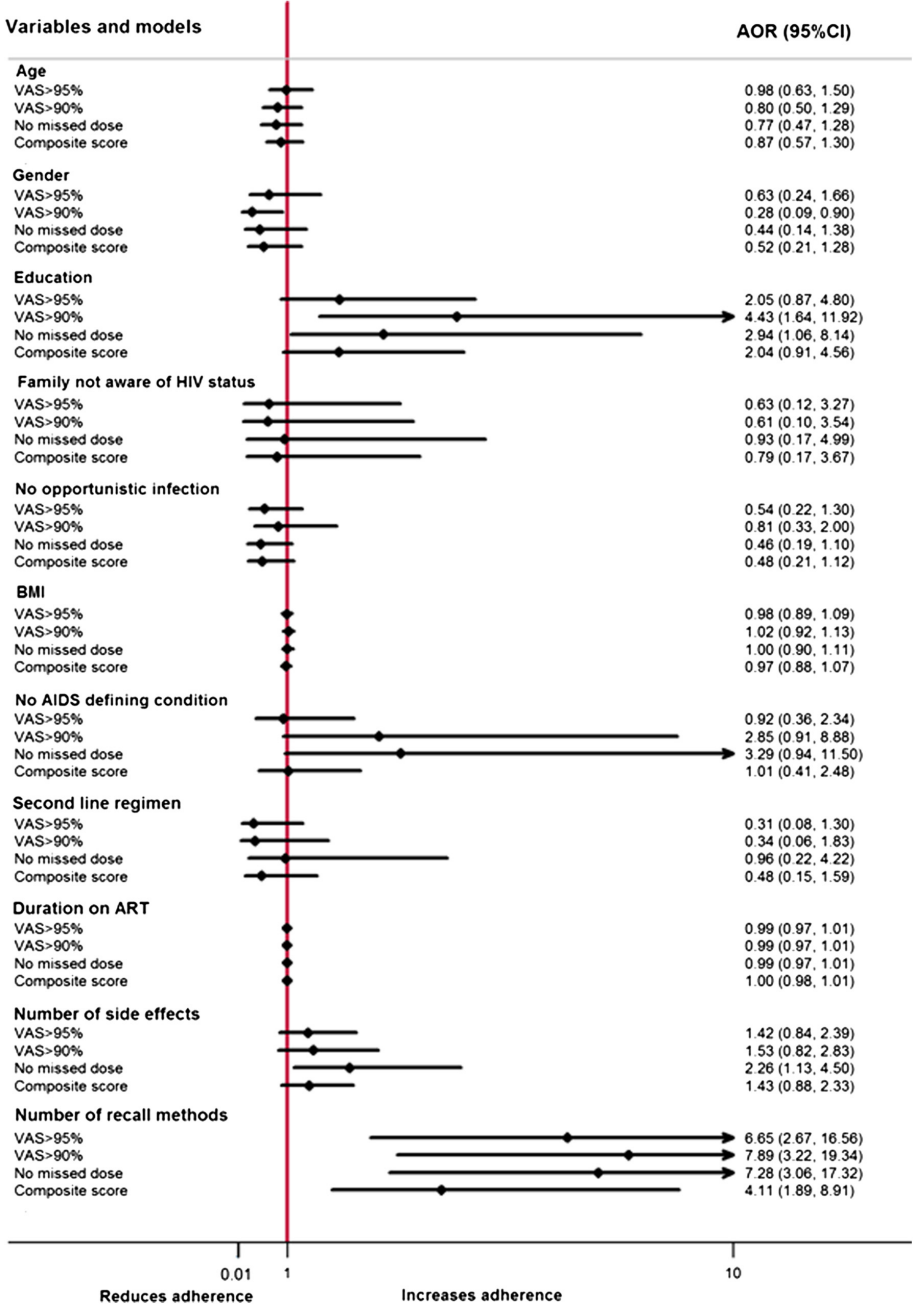
Multivariable analysis using different measures of adherence.

In the first model, we dichotomized adherence using the VAS at 95%. Using multiple recall methods was significantly associated with adherence >95% (AOR 6.7, 95% CI 2.69, 16.56; p<0.001). In our second model, adherence was dichotomized at 90%. Female gender was associated with adherence >90% (AOR 0.28, 95% CI 0.09, 0.90; p=0.032), while secondary education (AOR, 4.4 95% CI 1.64, 11.92; p= 0.003) and multiple recall methods (AOR, 7.89 95% CI 3.22, 19.33; p<0.001) were associated with adherence >90%. In the third model we used the number of missed doses as our measure of adherence. Experiencing more side effects (AOR 2.25, 95% CI 1.13, 4.50; p= 0.021) and using multiple recall methods increased the odds for not missing doses (AOR 7.28, 95% CI 3.06, 17.32; p<0.001). In our last model we used a composite measure of adherence that incorporated the VAS, number of missed doses and timing of doses. Only the use of multiple recall methods was associated with adherence (AOR 4.11, 95% CI 1.89, 8.91; p<0.001). The Hosmer and Lemeshow goodness-of-fit statistic for all the four models had p-values greater than 0.05 implying a good fit [42]. These models are displayed in Figure 2.

## Discussion

By reviewing the literature on adherence rates in Cameroon, and analysing a new data set we have produced an explanatory account of the trends in adherence observed and the reported determinants. We also identified another plausible means of enhancing adherence to ART: using multiple reminder methods.

Despite the different study designs, locations and sample sizes there is a clear improvement in adherence rates over time irrespective of how it is measured. This finding confirms and supports health policies that reduce the cost of care to improve access and use. This is also in line with studies reporting that the main hindrances to adherence in Africa are related to health system weaknesses such as inadequate supply, human resource shortages and poor infrastructure [29,32].

The determinants of adherence to ART identified in the Cameroonian literature are a subset of factors identified elsewhere [19]. However, there are discrepancies as to the role of monthly income and duration on ART on adherence. Boyer *et al*. [32] reported better adherence with a higher monthly income, while Rougemont *et al*. [31] reported the contrary. Both studies were initiated in the same period (2006) and therefore the cost of medication is unlikely to be the reason for this discrepancy. On the other hand Boyer *et al*. conducted a multisite (27 sites) study with a larger sample size (n=3151) compared to Rougemont *et al*. (n=312) in a single site. The results from Boyer *et al*. may be more plausible in the Cameroonian context where, apart from free ART, other related services are still funded by out-of-pocket payments and having to pay for care has been identified as a cause of non-adherence [25,28,29,32]. The larger sample is also more likely to have more accurate and generalizable data, especially since one of the sites included in Boyer *et al*. is the same site where the Rougemont study was conducted.

Roux *et al*. [30] noted a drop in adherence rates with increased duration on ART while Freeman *et al*. and Mbopi-Keou *et al*. [33,35] noted the contrary. However, these two samples differ greatly. Roux *et al*. describe a multisite study in the Centre region of Cameroon, in 401 PLHIV over two years. Freeman *et al*. describe a cross-national cohort of 8419 women from Cameroon, Burundi and the Democratic Republic of Congo followed up for two years. Female gender has been reported as a factor for better adherence [31] and this discrepancy may explain the interaction between gender and duration on medication. Mbopi-Keou *et al*. suggest that this trend might be the effect of continuous psycho-social support [33].

One study reported higher levels of adherence for patients who initiated ART with high levels of CD4-positive-T-lymphocytes [33]. This finding is in favor of earlier initiation of ART. Why patients who were transferred to the clinic would have better adherence rates is unclear. However, if they left their previous clinics due to service provision issues or unsatisfactory interactions with clinic staff, the latter clinic may provide a more favorable environment for adherence.

Using multivariable analysis we identified gender, education, side effects experienced, and number of reminder methods as factors that affect adherence rates.

The male gender has often been reported as the most likely to be non-adherent, maybe because males are more likely to engage in other behaviours that influence adherence such as binge drinking, tobacco use and drug use [19]. This effect was not found in all models and is not consistent across studies [19].

Level of education seems to play a role in adherence behaviour. A significant difference in adherence exists between people with secondary and those with no education. Further increments in educational level show no effect. This may imply that as concerns adherence to ART there are no benefits to be gained from very high levels of education. The benefits of education on adherence to ART can be obtained from secondary education. This effect was not consistent across all models.

Our findings regarding side effects are contrary to what is reported in literature. People who experienced more side-effects were more likely to be adherent. No other Cameroonian studies have identified side effects as a determinant for adherence to ART. In the CAMPS trial only 1% of those who missed doses reported side effects to be the reason for not taking medication (Table 3).

Only the number of reminder methods was associated with adherence in all the models. Another Cameroonian study reported the use of reminder methods to be associated with better adherence [30]. This is the first study to show that multiple reminders may have a cumulative effect. A randomized clinical trial in Kenya found alarms to have no effect on virological outcomes [43]. Simple electronic alarms are not as complex as the reminder methods described in this population (personal verbal reminders by individuals, phone alarms, meal times, timing with TV shows and watches). Other studies have reported the use of mobile phone beeps and prayer times by Muslims to remind them of when to take their medication [44]. The use of multiple and varied reminder methods may address other causes of non-adherence like forgetfulness and lack of social support.

The difference in the four models suggests that the factors associated with adherence depend somewhat on how adherence is defined and the thresholds for acceptable adherence. Different levels of adherence are associated with different factors. It is unclear how this issue can be resolved without a uniform and validated tool for measuring adherence rates in clinical practice or research, as all methods have their advantages and flaws [16,45]. Self reported adherence is by far the most popular method used, but how it is used varies greatly [16,46]. On the one hand, a simple visual analogue scale can be used to situate a patient’s adherence; and on the other a series of questions related to number of pills, timing, missed doses and identification of pills, all fall under the canopy of self report.

In low resource settings, self report, attendance-based and dispensing-based adherence measuring methods can predict important clinical outcomes [46] and should be collected routinely. Some measure of drug availability or the occurrence of drug stock-outs should be documented to explain trends in adherence behaviours.

Both sections of this paper may have limitations. Even though we observed an increasing trend in adherence as the cost of ART reduces, other temporal factors may contribute to this trend, notably health system improvements over the years and reductions in stigma and discrimination. These factors are reported as potential threats to optimal adherence and are very likely to change over time. The availability and use of reminder methods like mobile phones may also enhance adherence over time [25,32]. Participants in this study (CAMPS) may not adequately represent all the people living with HIV, but a subgroup who are already on ART, and who own mobile phones. Even though mobile phone ownership is widespread in Cameroon, those who own them may differ significantly from those who don’t.

## Conclusions

A prerequisite to optimal adherence is the availability of free or cheap medication. Irrespective of how it is measured, adherence rates tend to improve as the cost of medication is reduced. Improved financing mechanisms for ART are an important way of ensuring adequate adherence and improving outcomes for people living with HIV. Multiple and varied reminder methods are more likely to improve adherence than any single method alone. Further research is required to elucidate what combinations of reminder methods can produce the most desirable effects.

## Competing interests

The authors declare that they have no competing interests.

## Authors’ contributions

LM and LT conceived of the study; LM and SN performed the searches and extracted the data; LM, LT, SN, POZ, DY, LD and MS provided intellectual content and revised several versions of the manuscript. All authors read and approved the final manuscript.

## Funding

This study was funded in part by the CIHR Canadian HIV Trials Network (CTN).
